# Three-dimensional imaging and analysis of the internal structure of SAPO-34 zeolite crystals†

**DOI:** 10.1039/c8ra05918g

**Published:** 2018-10-01

**Authors:** Xue Bai, Bo Chen, Fei Yang, Xianping Liu, Daniel Silva-Nunes, Ian Robinson

**Affiliations:** School of Materials Science and Engineering, Tongji University Shanghai 201804 China bo.chen@tongji.edu.cn; London Centre for Nanotechnology, University College London London WC1H0AH UK i.robinson@ucl.ac.uk; Division of Condensed Matter Physics and Materials Science, Brookhaven National Laboratory Upton NY 11973 USA

## Abstract

SAPO-34 is widely used as a catalyst for important industrial reactions, such as the methanol-to-olefin (MTO) reaction and selective catalytic reduction (SCR) of nitrogen oxides (NO_*x*_). The internal structure of SAPO-34 has a great influence on the catalytic performance. Two-dimensional (2D) images of the SAPO-34 particle surfaces from scanning electron microscopy (SEM) show clearly well-faceted cube morphologies, which suggest they should be quite uniform perfect crystals. However, Bragg coherent X-ray diffraction imaging (BCDI) of the SAPO-34 particles shows a rich internal structure existing within these crystals. In this work, we investigated the internal structure of a SAPO-34 zeolite by serial block-face scanning electron microscopy (SBFSEM). The internal structure observed in the backscattered electron (BSE) micrographs from SBFSEM and the energy dispersive spectroscopy (EDS) measurements is found to be consistent with the BCDI results. From the three-dimensional (3D) structural images of SAPO-34 crystals obtained by SBFSEM, the domains within the individual SAPO-34 were visualized and quantified.

## Abstract 2

This work studies the inter-structure of a SAPO-34 particle by Bragg coherent X-ray diffraction imaging and serial-block-face scanning electron microscopy.

## Graphical abstract

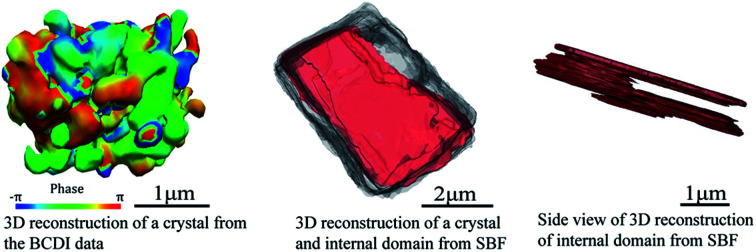

## 1. Introduction

Zeolites are of great scientific and technological importance because the petroleum-refining and petrochemical industries rely on their catalytic activity and shape selectivity.^1,2^ SAPO-34, a crystalline silico-aluminophosphate, is a member of the ALPOn zeolite family.^3^ Due to its unique microporous structure based on the chabazite (CHA) topology, SAPO-34 shows excellent catalytic selectivity and activity in the methanol-to-olefin (MTO) reaction,^4^ as well as in the selective catalytic reduction (SCR) of nitrogen oxides (NO_*x*_)^5^ and other reactions as well.^6^ The microporous SAPO-34 exhibits over 80% selective production of ethylene and propylene in MTO reactions with almost complete conversion of methanol.^7^ Hierarchical SAPO-34 zeolites, which were obtained by the introduction of secondary larger pores in the microporous SAPO-34 crystals to decreased the coke deposition, exhibit even better performance in the methanol-to-olefins (MTO) reaction.^7–9^ What's more, the Cu-SAPO-34 has been extensively used in the SCR of NO_*x*_ with ammonia (NH_3_) because of their high hydrothermal stability and its high efficiency to selectively reduce NO_*x*_ under the automotive conditions.^10,11^

To understand the catalytic properties and to gain an in-depth insight into the molecular uptake, intracrystalline diffusion and crystallization mechanisms, it is crucial to obtain fundamental knowledge about the internal structure, defects and distribution of elements.^12^ For example, the secondary system of large pores in the different hierarchical SAPO-34 treated with the different templates have a great influence on light olefin selectivity in the MTO reactions,^9^ and Gong *et al.* have proposed the crystallization mechanism of hierarchically organized SAPO-34 through the investigation of the internal structure and the formation process of the hollow SAPO-34 cubes.^8^ However, these researches reveal information from two-dimensional measurements instead of from three-dimensional (3D) investigation. Although, optical and interference microscopy were used to investigate the influence of internal crystal structure based on molecular uptake,^13,14^ as was revealed *in situ* mapping of the template-removal process in individual zeolite crystals using wide field and confocal fluorescence microscopy.^15^ There are reports on combination of focused ion beam (FIB) milling and electron backscattered diffraction (EBSD) imaging has also been applied to reveal the three-dimensional inner structure and phase orientation of zeolite crystals.^16^ Such kind of work on revealing 3D structure of SAPO-34 zeolites is still rarely reported. In the present work, the internal phases and cracks of the SAPO-34 crystals were demonstrated through a combination new approach: serial block-face scanning electron microscopy (SBFSEM) and Bragg coherent X-ray diffraction imaging (BCDI). This approach provides 3D information of the internal structure of SAPO-34 zeolite from the volumes much larger than that usually can be measured by FIB-SEM.^16^

BCDI is a unique imaging technique for nano- and micro-crystals, which utilizes both coherent X-ray sources and numerical algorithms.^17,18^ It has the ability of exploring the individual crystal diffraction and measuring strain and lattice displacements inside crystals.^19,20^ Through the phase retrieval algorithm, the density of the 3D image of a ZSM-5 zeolite crystal was reconstructed and an unusual internal deformation field distribution in ZSM-5 was tracked under different calcination conditions.^21^

SBFSEM is a destructive 3D imaging technique, which is based on *in situ* sectioning and imaging. The principle is to alternately image with a highly-sensitive back scattered electron (BSE) detector and cut with an ultra-microtome located within the SEM vacuum chamber. SBFSEM was first introduced by Kuzirian and Leighton^22,23^ and then largely-improved by Denk and Horstmann.^24^ Gatan company commercialized it under the name “3View”. It is exploited mostly for biological materials,^25,26^ light alloys,^27^ and organic coatings.^28^ Due to the SBFSEM method being much less used in inorganic crystalline materials, there are lots of challenges for using it on measuring SAPO-34 zeolite, such as possible imaging artefacts generated by epoxy and electron charging.^29^ In the work reported in this paper, AGAR 100 RESIN was modified with conductive carbon particles to reduce the charging effects while collecting BSE signals.

The BCDI result indicates that the SAPO-34 crystals have imperfect crystal structure consisting of internal domains. Here, SBFSEM investigation of the zeolite crystals, aiming at elucidating the microstructure inside the SAPO-34, is reported to show 3D grain structures. The BSE micrographs from both SBFSEM and the normal SEM, the energy dispersive spectroscopy (EDS) results of the polished sample and the three-dimensional (3D) crystalline microstructures reconstructed by Avizo illustrate different kinds of cracks and inner domains in the SAPO-34 particles, which is consistent with BCDI results. In addition, an improved sample preparation method is provided which will help using SBFSEM to measure other non-conductive inorganic hard condensed matters and materials in the future.

## 2. Experimental

### 2.1 Zeolite sample preparation

Samples of SAPO-34 powders with Si/P/Al = 0.4 : 1 : 1 were purchased from Nanjing JCNANO Tech Co., Ltd., Nanjing, China.

Samples for the normal BSE and EDS measurements were mechanically polished to bulk samples and then measured by a Zeiss Sigma 300 VP SEM compacted with an Oxford Aztec EDS (Carl Zeiss Microscopy Ltd., Cambridge, the UK and Oxford Instrument Ltd., Oxford, the UK).

Samples for SBFSEM measurements were embedded in carbon particles modified epoxy and trimmed by a Leica EM UC7 microtome. Unlike metals, inorganic crystals have low electrical conductivity as well as high hardness leading to difficulties for SBFSEM measurements. Although low-vacuum operation can reduce the charging,^24^ it will lose resolution under high magnification resulting in difficulty to distinguish the contrast of internal structure within the crystals. However, we found that graphite can not only improve the electrical properties of the polymer,^30^ but also has similar contrast as epoxy which can be easily distinguished from zeolite. 99% flake graphite (44 μm big) was therefore used to reduce the charging effect while doing SBFSEM measurements.

Conductive graphite powder, epoxy resin (Agar 100), curing agent (DDSA, MNA) and accelerator (BDMA) were mixed in the volume ratio of Agar 100: DDSA : MNA : BDMA = 20 : 12 : 9 : 1.2. Graphite powder (6.0 wt%) was dispersed in acetone in an ultrasonic bath for 2 h and under magnetic stirrer for 1 h. Then the epoxy resin was added into graphite–acetone mixture with 2 h of ultrasonic disperser (SKYMEN JP-020). The homogeneous mixture was then put in a vacuum oven at 60 °C overnight to remove the solvent. DDSA, MNA and BDMA were added to the mixture in turn and the mixture was shaken gently by hand with rotation for a few minutes. The zeolite was then embedded by mixing to prevent particle aggregation. The zeolite sample embedded in the epoxy resin mixture was finally cured in an oven for 24 h at 60 °C. After curing, the block was trimmed to a square block face approximately 0.50 mm × 0.50 mm using the microtome. Then the cubic block was glued onto a pin and painted with silver paint to connect it to the sample holder.

### 2.2 SBFSEM settings

The samples were imaged by a Zeiss Sigma 300 VP SEM, equipped with a Gatan 3View2XP system (Gatan Inc., Abingdon, the UK). The signal was collected using Gatan's BSE detector. The images were obtained in high vacuum mode (5.15 × 10^−7^ torr/6.87 × 10^−5^ Pa) at the acceleration voltage of 2 kV with a 2 μs per pixel dwell time under 15 μm aperture illumination. The block was sectioned with thickness step of 20 nm. Each image stack/volume contained 1024 × 1024 × 200 voxels with a voxel dimension of 24 nm (*x*-direction) × 24 nm (*y*-direction) × 20 nm (*z*-direction).

The serial images were collected in 16 bit format by Digital Micrograph software (Gatan Inc., the UK). Then Avizo was used to register the successive slices, segment the particles, generate 3D structural images and perform further quantitative analysis.

## 3. SBFSEM image analysis

### 3.1 Alignment of the sequence of images

The shifts between stacks caused by debris falling on the block face or electrostatic beam deflection would deform the real morphology of the sample.^24^ Hence, the images are aligned using the ‘Align Slices’ module in Avizo. Fig. S1 in ESI† shows a volume reconstruction of the same crystal particle before and after alignment, whose side length is 2.82 μm. After alignment, the *z* axis of the crystal has shifted from the slope to the vertical.

### 3.2 Segmentation of the particles

A 3D reconstruction (Fig. S2, ESI†) of the particles was segmented automatically by using Avizo. Automatic segmentation with the interactive thresholding module was used to get a binary image first. Then, the image was separated step-by-step by computing a distance map, creating markers from maximum regions of the distance map and applying the fast watershed algorithm module. The technique of fast watershed is based on a simulation of the rise of water from a set of markers.^31^ Different colors are randomly assigned to distinguish the different SAPO-34 particles by using “Labeling” module. In addition, through 3D length measurements in Avizo, the Feret length of the SAPO-34 particles were calculated and presented in Table S1 in the ESI.† It shows that the lengths of the particles are about 2 or 3 μm with one exception goes below 2 μm which is 1.7 μm big, and they have an average length of 2.7 μm. Although automatic segmentation is convenient and time-efficient, the resulted grain boundaries are coarse and could lead to inaccuracies in further quantitative analysis (Fig. S2, ESI†). Therefore, after automatic segmentation, manual segmentation was done to optimise the particle boundaries.

### 3.3 Filtering of small islands

Because the added conductive carbon lowers the adhesion of the original epoxy resin, during the cutting process, some small pieces of debris were deposited on the block surface rather than sticking on the knife surface. This results in the appearance of small islands after reconstruction (Fig. S3B, ESI†). Fig. S3A in ESI† shows a 3D volume rendering containing SAPO-34 crystals coloured in red and small islands coloured in dark blue (see Fig. S3B† as well). Using Avizo's ‘Label Analysis Filter’ module, the small islands (in Fig. S3A†) whose volume was less than 0.56 μm^3^ which has 4.9 × 10^4^ voxels (roughly a 36 × 36 × 36 voxel cube) were removed. The total volume fraction of small islands was 1.4%.

## 4. Results and discussions

### 4.1 X-ray diffraction (XRD) and SEM result of the SAPO-34 powder

The powder XRD pattern of the sample (Fig. S4, ESI†) is in good agreement with typical diffraction pattern of the CHA structure belonging to the *Rm* space group.

**Fig. 1:**
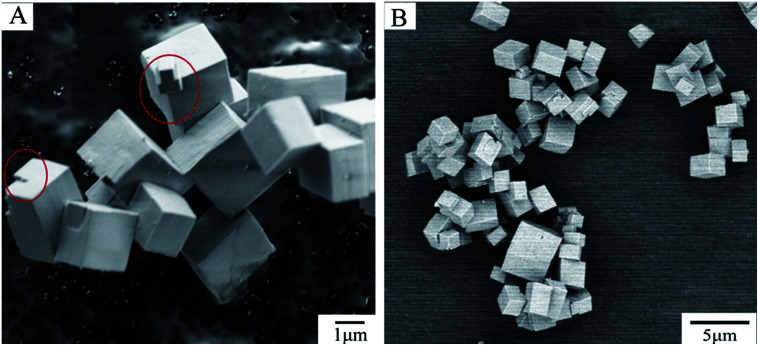
SE and BSE images of SAPO-34 powder (A) SE image of SAPO-34 powder measured at 1 kV. The regions within the red circles represent the small kinks and steps on the surface of sample. (B) BSE image of SAPO-34 powder measured at 2 kV from SBFSEM.

Fig. 1 shows secondary electron (SE) (Fig. 1A) and BSE (Fig. 1B) micrographs of SAPO-34. It is clear that the samples have well-formed cubic morphology with the regular cube or rectangular shape typical of the CHA structure as reported in previous literature.^32^ The surface of sample appears smooth, with only a small amount of kinks and steps (see red circle in Fig. 1A).

### 4.2 BCDI result

**Fig. 2:**
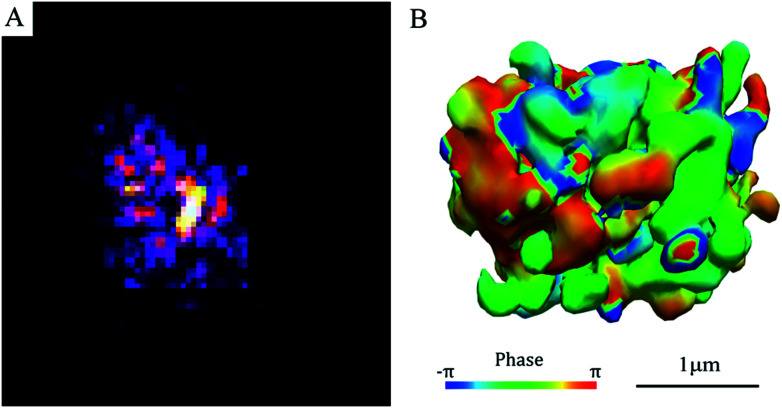
Diffraction pattern and iso-surface of a single SAPO-34 particle by BCDI (A) one slice of the Bragg coherent diffraction pattern (B) iso-surface view of a 3D reconstruction of the BCDI data, with the surface coloured by the phase of the 3D image.

Fig. 2A exhibits the diffraction pattern of a SAPO-34 crystal obtained by BCDI at the coherence branch of the I-13 beamline of Diamond Light Source (DLS) in the UK. It shows an asymmetric diffraction pattern with speckles surrounding a single-centred Bragg peak. Because it is single-centred, this allows the inversion of the diffraction pattern to an image with standard support-based algorithms.^18^ The 3D diffraction data were inverted to real space images by a sequence of hybrid input–output and error reduction algorithms. The iso-surface of the resulting density of the 3D image of the SAPO-34 crystal is shown in Fig. 2B. The shape appears to be an irregular cube with a rough-surface and many internal domains with different phases shown in colours on their surfaces in Fig. 2B. This is quite different from the SEM image shown in Fig. 1A. We conclude there is an internal domain structure inside the SAPO-34 crystal “visible” to coherent X-ray diffraction, but “invisible” in 2D SE micrographs. Such a phase domain structure implies there must be a network of zero-angle grain boundaries inside the crystal, isolating domains of the same orientation but slightly offset positions.

### 4.3 2D images of SAPO-34 by SBFSEM and BSE/EDS

**Fig. 3:**
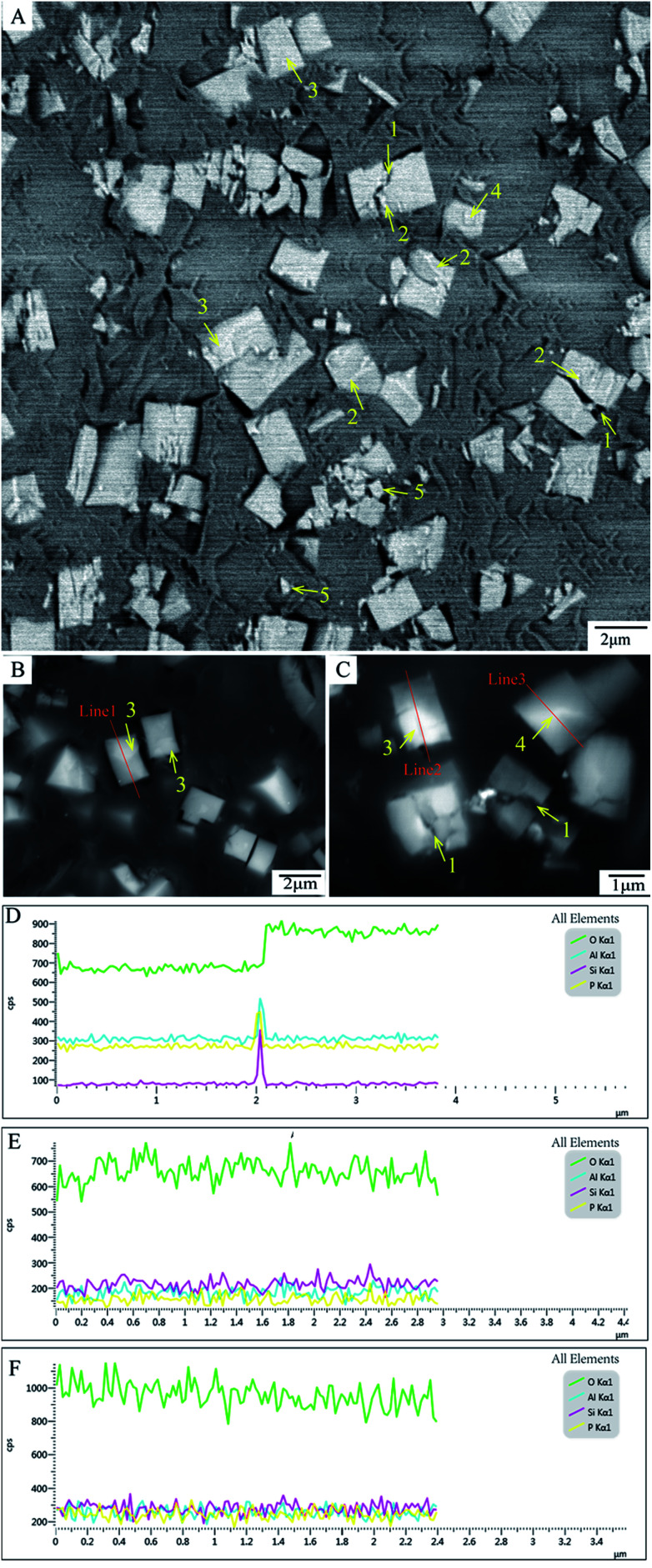
2D images of SAPO-34 from SBFSEM and BSE/EDS (A) cross-section of series images of SBFSEM micrographs (2 kV accelerating voltage and 20 nm slicing thicknesses); (B and C) BSE images of the polished flat surface of the SAPO-34 embedded resin block (20 kV accelerating voltage); (D, E and F) Representative line scan of line 1, 2, 3 using EDS; features 1–5 pointed by yellow arrow are respectively cracks, impurities, the first kind of domains, the second kind of domains and small debris.

The “cracks” within SAPO-34 are seen both in features 1 pointed by yellow arrows in the SBFSEM slice (Fig. 3A) and features 1 in the normal BSE image (Fig. 3C), and most of them are in the middle of crystal. The presence of impurities, another kind of structure within the SAPO-34 crystals, which look like brighter strips at the edge of each crack or in the particles, are shown as the features 2 (pointed by yellow arrows as well) in Fig. 3A. Impurity phase formation is possibly caused by the appearance of cristobalite, a dense phase of aluminophosphate structure. It is reported that solutions may be formed with silicon atoms below a threshold level, which tends to produce an impurity phase upon crystallization of SAPO-34.^33^ Impurities would lead to tensile strain incorporated in the crystal matrix during crystallization and the resulting stress may give rise to crack formation and could also cause the array of phase domains seen in the BCDI. After the final crystallization, tensile strain also remained in crystal. As a result, the cracks would propagate inside the growing crystal. During cutting process in SBFSEM, these cracks will preferentially fracture. The cracks, a kind of the internal structure of SAPO-34, indicate that the regions surround them have impurities and defects and could change the surface area of the SAPO-34 particles. This could increase their molecular uptake and catalytic properties.^12^

Inner domains of SAPO-34 crystals such as features 3 and 4 in Fig. 3A–C could be seen, which appear with brighter contrast. Two distinct kinds of domains can be identified: the first kind showing as features 3 in Fig. 3A–C, which resemble rectangular shapes close to the edges of the crystals. Whereas in the second example shows as features 4 in Fig. 3A and C. These domains have irregular shapes in the middle of the crystals.

To find out whether other chemical elements are existing in the crystal domains and to look for the fluctuation of elemental ratio associated with the domains, EDS line scans 1, 2 and 3 of the different domains are plotted, and the results are shown in Fig. 3D, E and F respectively. It is clear that these domains contain O, Al, Si and P elements. It can be observed that the proportion of oxygen is increased at the edge of the square-shape domain scanned by ‘Line 1’, as is shown in Fig. 3D. The domain scanned by ‘Line 2’ and the domain scanned by ‘Line 3’ have no significant chemical fluctuation shown in Fig. 3E and F, respectively. So the formation of these domains are not due to measurable compositional changes, but may be caused by different crystal orientation.

Although the modified Agar 100 epoxy resin reduced the charging effect, it was still found to have some artefacts. Features 5 in Fig. 3A represent small pieces of debris dropped on the open block-face which become small islands after 3D rendering (see Fig. S3B, ESI†), as mentioned previously as well. Currently, in order to make an embedding matrix having good embedding and cutting performance, efforts are under way to find better modifications to the epoxy resin using other conductive particles.

### 4.4 Three- dimensional reconstruction of SAPO-34 crystal

**Fig. 4:**
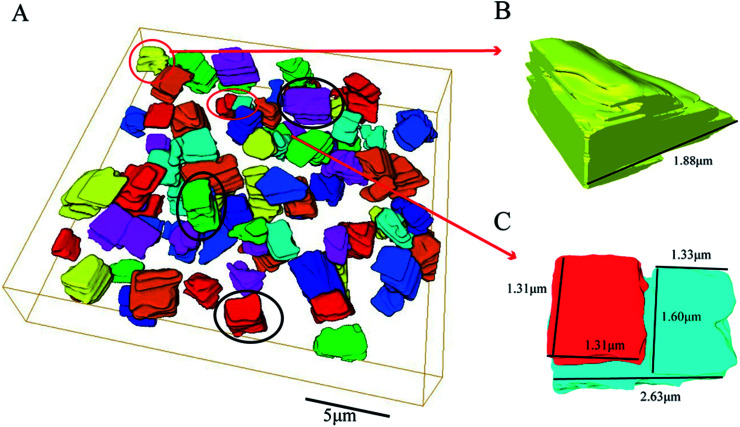
A volume reconstruction of SAPO-34 obtained by semi-automatic segmentation (A) 3D reconstruction of a volume of the SAPO-34 sample (B and C) Two kinds of reconstruction of incomplete single particles.

**Fig. 5:**
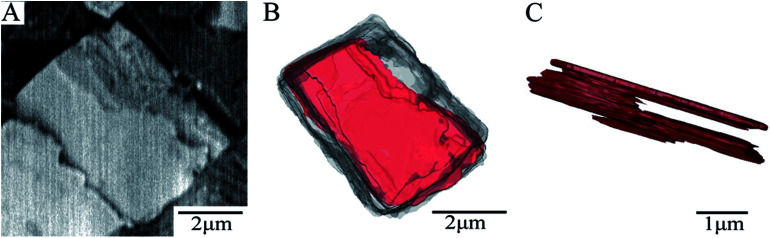
BSE image and 3D reconstruction of a single SAPO-34 particle (A) a crop from the BSE cross-section of series images of SBFSEM micrographs (2 kV accelerating voltage and 20 nm slicing thicknesses); (B) 3D reconstruction of a crystal and internal domain (C) the side view of 3D reconstruction of internal domain.

The morphology of the whole SAPO-34 crystal sample, shown in Fig. 4, was reconstructed from the obtained series of BSE micrograph slices from SBFSEM by Avizo. Most particles are complete and have cubic-like morphology (see particles in the black circles in Fig. 4A) which are basically consistent with SEM results in Fig. 1. A single complete particle was reconstructed separately, and is shown in Fig. 5B. Fig. 5A represents an intermediate slice in the sequence of SBFSEM images, in which internal domains with brighter contrast can be clearly observed. As is shown in Fig. 5B, the grey and red colours represent the full 3D rendering of a whole single particle and the internal domain in it, respectively. The red part in Fig. 5B and C was the 3D reconstruction of the lighter domain inside the crystal in Fig. 5A. The shape of the internal domain is close to a rectangular shape, located near the edge of the crystal. The volume of the domain occupies about 29% of the whole crystal. In Fig. 5C, the other parts have been removed from the view to display only the domain. The upper region is relatively large, while the middle part becomes smaller and then the bottom part becomes larger again. Finally, the domain location changes from the crystal edge to the middle of the crystal grain, which indicate that the domain, in three dimensions, changes from the domain such as feature 3 in Fig. 3 to the domain such as feature 4 in Fig. 3. These complex internal structure is consistent with the results from the BCDI diffraction patterns.

Due to the limitation of the experiment conditions, there are two kinds of particles which are different from complete particles in the SBFSEM images. They are shown in Fig. 4B and C. In Fig. 4B, this is an incomplete particle at the edge of the whole reconstruction volume due to the selection of imaging field of view. Secondly, Fig. 4C shows a particle which becomes split into two parts over its depth. The length of whole cube is 2.63 μm. The length and the width of red “broken” part is both 1.31 μm. The length and the width of upper blue part are 1.33 and 1.60 μm, respectively. From which, we could know that the two parts are almost evenly split.

## 5. Conclusions

A single particle of SAPO-34 zeolite was investigated by BCDI. A complicated single-centred diffraction pattern from BCDI indicates SAPO-34 has internal domain structures, which are hidden from the simple cube shapes observed by 2D SEM observation. By means of the combination of cross-sectional images from SBFSEM and normal BSE & EDS measurements, internal cracks, impurity and domains and chemical distribution of SAPO-34 were observed, which is consistent with the BCDI investigation results. It was demonstrated that the most of internal cracks of sample are located at the middle of crystal and may be caused by a stripe-like impurity in the crystals. The different domains within the SAPO-34 was revealed. The formation of these domains may be caused by oxygen elemental changes or different crystal orientation. In addition, the 3D microstructure of a single SAPO-34 grain was reconstructed from the sequence images of SBFSEM to investigate 3D morphology of the internal domains of SAPO-34 crystals. This information cannot be obtained from traditionally 2D imaging techniques such as the normal SEM.

The successful demonstration of SBFSEM applied to SAPO-34 crystals shows that SBFSEM could be a suitable method to study 3D internal nano- and micro- structure of zeolite crystals. It provides a new approach to give better understanding of the connection between the catalytic properties and internal structure of these materials.

## Conflicts of interest

There are no conflicts to declare.

## Supplementary Material

RA-008-C8RA05918G-s001

## References

[cit1] Silva A. O. S., Souza M. J. B., Arajuo A. S. (2001). React. Kinet. Catal. Lett..

[cit2] Porcelli J. V. (2000). Chem. Eng. Prog..

[cit3] Lok B. M. , Messina C. A., Patton R. L., Gajek R. T., Cannan T. R. and Flanigen E. M., *US Pat.*, 4440871, 1984

[cit4] Zhu Z. D., Hartmann A. M., Larry K. (2000). Chem. Mater..

[cit5] Wang L., Li W., Qi G., Duan W. (2012). J. Catal..

[cit6] Shi J., Wang Y., Yang W., Tang Y., Xie Z. (2016). Chem. Soc. Rev..

[cit7] Sun Q., Xie Z., Yu J. (2018). Natl. Sci. Rev..

[cit8] Gong J., Tong F., Ji X., Zeng C., Wang C., Lv Y., Zhang L. (2014). Cryst. Growth Des..

[cit9] Chen X., Xi D., Sun Q., Wang N., Dai Z., Fan D., Valtchev V., Yu J. (2016). Microporous Mesoporous Mater..

[cit10] Ma L., Cheng Y., Cavataio G., Mccabe R. W., Fu L., Li J. (2013). Chem. Eng. J..

[cit11] Yu T., Fan D., Hao T., Wang J., Shen M., Li W. (2014). Chem. Eng. J..

[cit12] Muller G., Narbeshuber T., Mirthand G., Lercher J. A. (1994). J. Phys. Chem..

[cit13] Lehmann E., Chmelik C., Scheidt H., Vasenkov S., Staudte B., Karger J., Kremer F., Zadrozna G., Kornatowski J. (2002). J. Am. Chem. Soc..

[cit14] Kocirik M., Kornatowski J., Masarik V., Novak P., Zikanova A., Maixner J. (1998). Microporous Mesoporous Mater..

[cit15] Karwacki L., Stavitski E., Kox M. H. F., Kornatowski J., Weckhuysen B. M. (2008). Stud. Surf. Sci. Catal..

[cit16] Stavitski E., Drury M. R., de Winter D. A., Kox M. H., Weckhuysen B. M. (2010). Angew. Chem..

[cit17] Williams G. J., Pfeifer M. A., Vartanyants I. A., Robinson I. K. (2003). Phys. Rev. Lett..

[cit18] Ulvestad A., Cho H. M., Harder R., Kim J. W., Dietze S. H., Fohtung E., Meng Y. S., Shpyrko O. G. (2014). Appl. Phys. Lett..

[cit19] Robinson I. K., Harder R. (2009). Nat. Mater..

[cit20] Newton M. C., Leake S. J., Harder R., Robinson I. K. (2010). Nat. Mater..

[cit21] Cha W., Jeong N. C., Song S., Park H. J., Thanh P. T. C., Harder R., Lim B., Xiong G., Ahn D., McNulty I., Kim J., Yoon K. B., Robinson I. K., Kim H. (2013). Nat. Mater..

[cit22] Kuzirian A. M., Leighton S. B. (1983). Scanning Electron Microsc..

[cit23] Leighton S. B. (1981). Electron Microsc..

[cit24] Denk W., Horstmann H. (2004). PLoS Biol..

[cit25] Chen B., Yusuf M., Hashimoto T., Estandarte A. K., Thompson G., Robinson I. (2017). Sci. Adv..

[cit26] Giacci M. K., Bartlett C. A., Huynh M., Kilburn M. R., Dunlo S. A., Fitzgerald M. (2018). Sci. Rep..

[cit27] Thompson G. E., Hashimoto T., Zhong X. L., Curioni M., Zhou X., Skeldon P., Withers P. J., Carr J. A., Monteith A. G. (2013). Surf. Interface Anal..

[cit28] Chen B., Sicairos M. G., Xiong G., Shemilt L., Diaz A., Nutter J., Burdet N., Huo S., Mancuso J., Monteith A., Vergeer F., Burgess A., Robinson I. K. (2013). Sci. Rep..

[cit29] Hashimoto T., Thompson G. E., Zhou X., Whiters P. J. (2016). Ultramicroscopy.

[cit30] Tchmutin I. A., Ponomarenko A. T., Krinichnaya E. P., Kozub G. I., Efimov O. N. (2003). Carbon.

[cit31] Cocks E., Taggart M., Rind F. C., White K. (2018). J. Microsc..

[cit32] Dahl I. M., Wendelbo R., Andersen A., Akporiaye D., Mostad H., Fuglerud T. (1999). Microporous Mesoporous Mater..

[cit33] Shalmani F. M., Halladj R., Askari S. (2012). Powder Technol..

