# Supplementary material for: Three-dimensional imaging and analysis of the internal structure of SAPO-34 zeolite crystals
Source: RSC Adv. 2018 Oct 1;8(59):33631–6. doi: 10.1039/c8ra05918g (PMC9086581; doi:10.1039/c8ra05918g)
Supplement: RA-008-C8RA05918G-s001 [file RA-008-C8RA05918G-s001.pdf]

Electronic Supplementary Material (ESI) for RSC Advances.
This journal is © The Royal Society of Chemistry 2018

Supplementary Information

Three-dimensional imaging and analysis of internal structure of SAPO-34 zeolite crystals

Xue Bai[a], Bo Chen[a,b,*], Fei Yang[a], Xianping Liu[a], Daniel Silva-Nunes[c] and Ian Robinson[a,b,c,*]

[a] School of Materials Science and Engineering, Tongji University, Shanghai 201804, China.E-mail: bo.chen@tongji.edu.cn

[b] London Centre for Nanotechnology, University College London, London WC1H0AH, U.K. E-mail: i.robinson@ucl.ac.uk

[c] Division of Condensed Matter Physics and Materials Science, Brookhaven National Laboratory, Upton NY 11973, USA

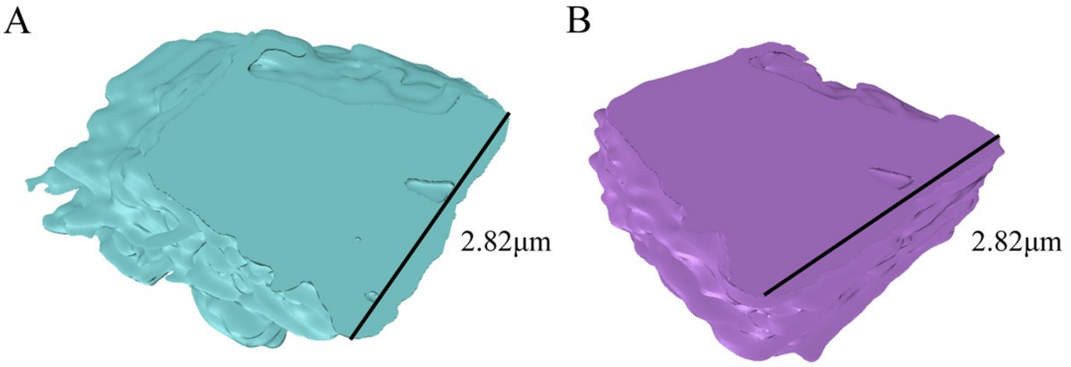

Figure S1 3D rendering of a single SAPO-34 particle. A: 3D images of the SAPO-34 particle before the alignment treatment of the slices. B: 3D images of the SAPO-34 particle after the alignment.

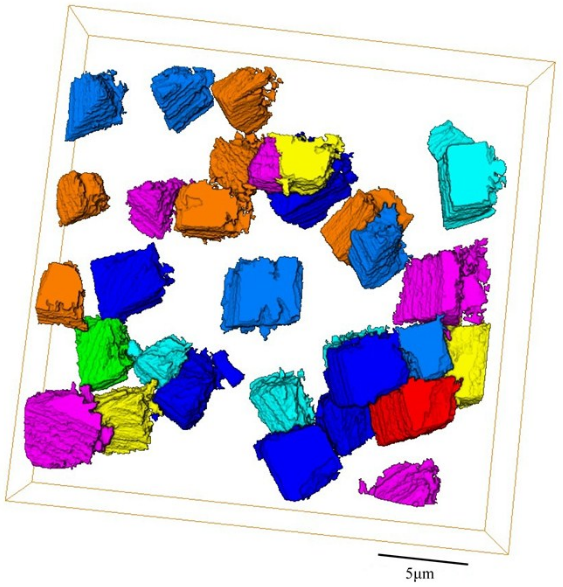

Figure S2 Volume reconstruction obtained by automatic segmentation. (Colors are randomly assigned to each SAPO-34 particle by labeling module of Avizo software.)

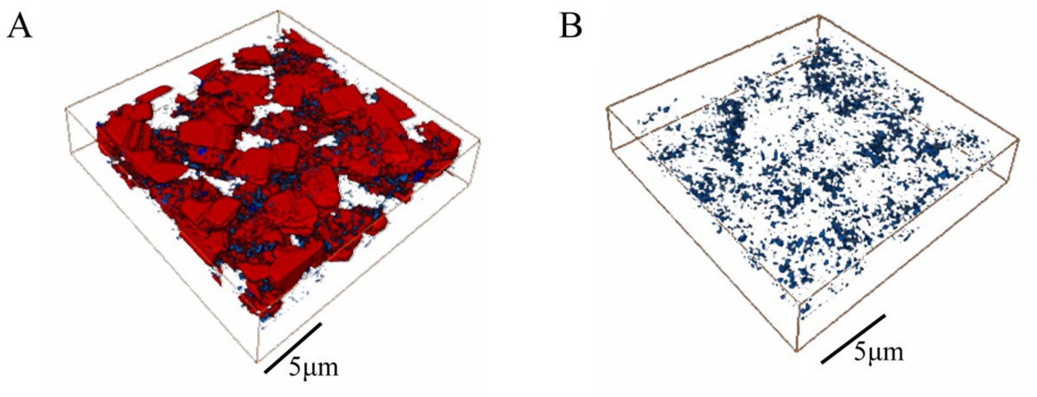

Figure S3 Volume rendering of particles and small islands. A: particles (red) and small islands (blue) B: small islands alone

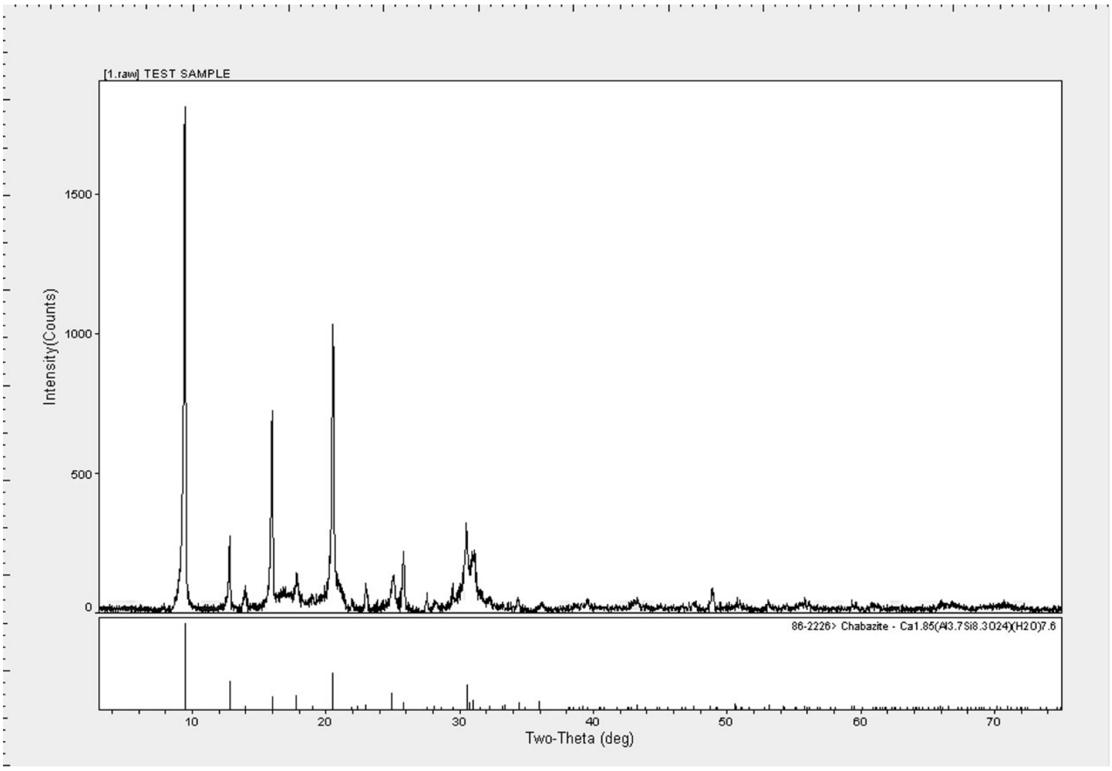

Figure S4 Powder X-ray Diffraction (XRD) pattern of the SAPO-34 sample

Table S1 Feret lengths of the observed 27 SAPO-34 obtained from Avizo software after segmentation

Number	Length3D(μm)	Number	Length3D(μm)
1	3.581	15	2.522
2	3.494	16	2.516
3	3.407	17	2.443
4	3.185	18	2.419
5	3.180	19	2.364
6	3.113	20	2.294
7	3.107	21	2.244
8	3.106	22	2.244
9	3.086	23	2.189
10	2.902	24	2.164
11	2.875	25	2.140
12	2.816	26	2.032
13	2.775	27	1.734
14	2.652	Mean	2.688
